# Cytokine Profiling in Cerebrospinal Fluid of Patients with Newly Diagnosed Relapsing-Remitting Multiple Sclerosis (RRMS): Associations between Inflammatory Biomarkers and Disease Activity

**DOI:** 10.3390/ijms25137399

**Published:** 2024-07-05

**Authors:** Barbara Gębka-Kępińska, Bożena Adamczyk, Dorota Gębka, Zenon Czuba, Jarosław Szczygieł, Monika Adamczyk-Sowa

**Affiliations:** 1 Department of Neurology, Faculty of Medical Sciences in Zabrze, Medical University of Silesia, 41-800 Katowice, Poland; barbara.gebka@gmail.com (B.G.-K.); bozena.m.adamczyk@gmail.com (B.A.); dorota.gebka8@gmail.com (D.G.); jwszczygiel@gmail.com (J.S.); 2 Department of Microbiology and Immunology, Faculty of Medical Sciences in Zabrze, Medical University of Silesia, 41-800 Katowice, Poland; zczuba@sum.edu.pl

**Keywords:** multiple sclerosis, cytokines, immune system, biomarkers, pathomechanism, CSF

## Abstract

Cytokines regulate immune responses and are crucial to MS pathogenesis. This study evaluated pro-inflammatory and anti-inflammatory cytokine concentrations in the CSF of de novo diagnosed RRMS patients compared to healthy controls. We assessed cytokine levels in the CSF of 118 de novo diagnosed RRMS patients and 112 controls, analyzing relationships with time from symptom onset to diagnosis, MRI lesions, and serum vitamin D levels. Elevated levels of IL-2, IL-4, IL-6, IL-13, FGF-basic, and GM-CSF, and lower levels of IL-1β, IL-1RA, IL-5, IL-7, IL-9, IL-10, IL-12p70, IL-15, G-CSF, PDGF-bb, and VEGF were observed in RRMS patients compared to controls. IL-2, IL-4, IL-12p70, PDGF, G-CSF, GM-CSF, and FGF-basic levels increased over time, while IL-10 decreased. IL-1β, IL-1RA, IL-6, TNF-α, and PDGF-bb levels negatively correlated with serum vitamin D. TNF-α levels positively correlated with post-contrast-enhancing brain lesions. IL-15 levels negatively correlated with T2 and Gd(+) lesions in C-spine MRI, while TNF-α, PDGF-bb, and FGF-basic correlated positively with T2 lesions in C-spine MRI. IL-6 levels positively correlated with post-contrast-enhancing lesions in Th-spine MRI. Distinct cytokine profiles in the CSF of de novo diagnosed MS patients provide insights into MS pathogenesis and guide immunomodulatory therapy strategies.

## 1. Introduction

Multiple sclerosis (MS—sclerosis multiplex) is a chronic autoimmune inflammatory disease of the CNS, showing the occurrence of demyelination with axonal damage, limited remyelination, oligodendrocyte death, gliosis (scarring), and neurodegeneration. The disease is characterized by diffuse, multifocal damage to the central nervous system, both the white and gray matter of the brain and the spinal cord [1]. The latest data from the Multiple Sclerosis Atlas, a joint project of the International Multiple Sclerosis Federation and the WHO, show that 2.9 million people worldwide suffer from MS [2]. The disease affects mainly young adults, aged between 18 and 40 years, with whom chronic buildup of physical and cognitive disability has a significant impact on their social, economic, and individual well-being. MS is the most common non-traumatic cause of neurological disability in this age group. Clinical manifestations of MS depend on the location of the CNS lesions. The symptoms may include sensory and visual disturbances; motor and coordination impairment; as well as spasticity, fatigue, pain, and cognitive deficits [3]. The disease may develop as a clinically isolated syndrome (CIS), while about 85–90% of patients develop then a relapsing-remission form of the disease, characterized by periods of worsening symptoms followed by remission. As the disease progresses, the recovery is incomplete, and about 50% of the patients develop eventually a form of the disease known as secondary progressive MS, characterized by progressive, irreversible accumulation of neurological disability. A smaller number of patients with MS (10–15%) follow a progressive clinical course from the onset of the disease, which is referred to as primary progressive MS [4]. The exact etiology of the disease remains unknown. Numerous studies indicate that a complex interaction between environmental factors and genetic susceptibility underlies its immunopathogenesis. Immune mechanisms have a key role in the pathogenesis of MS. Traditionally, multiple sclerosis has been considered an autoimmune disease mediated by T lymphocytes. The role of TCD4+ lymphocytes and TCD8+ lymphocytes, which are the main cells crossing the blood–brain barrier, is to recognize the nerve tissue autoantigens, most likely derived from myelin proteins, and differentiate into Th1, Th2, and Th17 helper lymphocytes. Th1, Th2, and Th17 lymphocytes damage the CNS structures directly or indirectly through pro-inflammatory cytokines (IFN-γ, TNF-α, IL-2, IL-12, IL-15, IL-17, IL-21, and IL-22) [5].

B lymphocytes have key functions in the immune response, including responsibility for antibody production, antigen presentation, and cytokine secretion. In multiple sclerosis, attention is now turning to the function of B lymphocytes independent of antibody production. In the course of MS, they show an altered cytokine production profile, with the predominance of pro-inflammatory factors. The production capacity of anti-inflammatory IL-10 by B lymphocytes decreases, while secretion of pro-inflammatory TNF-α increases, leading to oligodendrocyte damage [6].

Cytokines are involved in the regulation of the immune response in multiple sclerosis (MS). From activation and differentiation of T lymphocytes, promotion of the inflammatory response, and induction of myelin sheath damage to modulation of repair processes, cytokines play a key role in many aspects of MS pathogenesis [7].

It is now also believed that abnormal signal exchange occurs between TCD4+ lymphocytes, TCD8+ lymphocytes, B lymphocytes, myeloid cells, and the central nervous system (CSN) resident cell populations such as microglia cells and astrocytes, possibly resulting in the induction of CNS degeneration [8].

The purpose of this study is to evaluate the concentrations of IL-1β, IL-1RA, IL-2, IL-4, IL-5, IL-6, IL-7, IL-8, IL-9, IL-10, IL-12p70, IL-13, IL-15, IL-17A, TNF-α, VEGF, PDGF-bb, GM-CSF, G-CSF, and FGF-basic in the CSF of patients with newly diagnosed relapsing-remitting multiple sclerosis (RRMS) and a group of healthy control subjects without demyelinating brain lesions. Table 1 shows the available literature data on the nature of each cytokine. In addition, we evaluated possible correlations between the levels of pro-inflammatory and anti-inflammatory cytokines according to age, time span between the first symptoms and diagnosis, radiological parameters, and serum vitamin D levels. This study is innovative, evaluating the usefulness of new markers in the diagnostic process of RRMS.

## 2. Results

The study group and the control were dominated by females, accounting for 73% and 79%, respectively. Both groups were homogeneous in terms of age. The median age at the time of MS diagnosis was 38.6 years, and the median time between the first symptoms and the diagnosis was 5.15 years.

The median number of T2-weighted lesions on brain MRI for the RRMS group was 16.65, and the median number of enhancing lesions after contrast on brain MRI was 0.90. The median number of T2-weighted lesions on MRI of the cervical spine was 5.54, and the median number of active lesions on MRI of the cervical spine was 0.35. The median number of T2-weighted lesions on MRI of the thoracic spine was 4.35, and the median number of active lesions on MRI of the thoracic spine was 0.23.

In the study group, the presence of type 2 oligoclonal striations was demonstrated in 74.23% of patients. The motor disability of patients with newly diagnosed RRMS was assessed using the Expanded Disability Status Scale (EDSS), with a mean score of 1.8 points (Table 2).

### 2.1. Analysis of Concentrations of Selected Interleukins in the Cerebrospinal Fluid in Patients with Newly Diagnosed RRMS and in the Controls

The study group showed statistically higher concentrations of IL-2, IL-4, IL-6, IL-13, FGF basic, and GM-CSF in the CSF as compared to concentrations of these cytokines in the CSF of the Control group (Mann–Whitney U test *p* < 0.0001). The results also showed statistically lower concentrations of the cytokines IL-1b, IL-1RA, IL-5, IL-7, IL-9, IL-10, IL-12p70, IL-15, G-CSF, PDGF-bb, and VEGF in the CSF as compared to concentrations of these cytokines in the CSF of the Control group (Mann–Whitney U test *p* < 0.0001). The concentrations of IL-8, IL-17A, and TNFα in the CSF showed no statistical relationship in the study group as compared to the controls. The above data are shown in Table 3.

### 2.2. Correlations of Selected Interleukins in the Cerebrospinal Fluid with Serum Vitamin D Levels in the Study and Control Groups

In patients with newly diagnosed RRMS, IL-1B, IL-1RA, IL-6, TNFα, and PDGF-bb levels in CSF correlated negatively with serum vitamin D levels (Table 4).

### 2.3. Correlations of Selected Interleukins in the Cerebrospinal Fluid with Duration of Disease in the Study Group (with Time from the Onset of the First Symptoms to Diagnosis)

In patients with newly diagnosed RRMS, CSF levels of Il-2, IL-4, IL-12p70, PDGF-bb, GM-CSF, G-CSF, and FGF basic correlated positively with the time from the onset of the first symptoms to the diagnosis. The concentration of IL-10 in the CSF showed a negative correlation with the time from the onset of the first symptoms to diagnosis (Table 5).

### 2.4. Correlations of Selected Interleukins in the Cerebrospinal Fluid with the Number of T2 and Gd(+) Lesions on MRI

We found no correlation between the selected cytokines in CSF and the number of T2 lesions on brain MRI, while TNFα levels in CSF correlated positively with the number of post-contrast-enhancing lesions within the brain (Table 6). In patients with newly diagnosed RRMS, IL-15 levels in CSF correlated negatively with both the number of T2 lesions in C spine MRI and the number of Gd(+) lesions in C spine MRI, while TNFα, PDGF-bb, and FGF basic in CSF correlated positively with the number of T2 lesions in C spine MRI (Table 7). No correlation was found between selected cytokines in CSF and the number of T2 lesions in Th spine MRI, while IL-6 levels in CSF correlated positively with the number of post-contrast enhancing lesions in Th spine MRI (Table 8).

## 3. Discussion

Cytokines are involved in the immune response and may influence the course of the disease in multiple sclerosis. Cytokines function as signaling molecules within the immune system, modulating the immune responses and facilitating either pro-inflammatory or anti-inflammatory activities. In multiple sclerosis, any imbalance between pro-inflammatory and anti-inflammatory cytokines can affect the severity and progression of the disease [10].

Our study showed that both interleukin-1 beta (IL-1β) and interleukin-1 receptor antagonist (IL-1Ra) levels in CSF were significantly lower in patients with newly diagnosed relapsing-remitting multiple sclerosis (RRMS) than in healthy controls. In addition, we found a negative correlation between IL-1β levels in CSF and serum vitamin D levels, while IL-1Ra showed a positive correlation with serum vitamin D levels.

Evidence suggests that vitamin D can affect the expression and activity of IL-1β and IL-1Ra, as supported by numerous studies showing that adequate vitamin D levels correlate with reduced IL-1β production in various cell types, including human monocytes and macrophages. Moreover, in the context of autoimmune diseases such as multiple sclerosis, higher levels of vitamin D are associated with reduced disease activity and reduced levels of pro-inflammatory cytokines, including IL-1β [11,12,13]. Our analyses highlight the potential role of IL-1Ra in regulating the inflammatory response and suggest that low levels of IL-1Ra may be associated with vitamin D deficiency, which may affect the course and progression of RRMS.

Irena Dujmovic and colleagues, examining the cerebrospinal fluid of patients with multiple sclerosis during relapse before the administration of steroid therapy, showed significantly elevated levels of IL-1β, IL-1Ra, and Acp in the cerebrospinal fluid of patients with multiple sclerosis compared to controls [14].

In contrast, a study by Silvia Rossi and colleagues showed that the IL-1β/IL-1Ra ratio was significantly higher in the cerebrospinal fluid of patients with active multiple sclerosis [15].

Our study focused on patients with newly diagnosed RRMS who were not receiving immunomodulatory treatment and had not experienced a relapse. Cytokine levels may vary due to progressive inflammation and the immunotherapy used. These findings also suggest that we may observe dysregulation of the interleukin-1 (IL-1) pathway in CSF in patients with de novo diagnosed multiple sclerosis.

We showed that IL-2 levels in the cerebrospinal fluid of patients with newly diagnosed relapsing-remitting multiple sclerosis (RRMS) were significantly higher than those of healthy controls. Furthermore, we showed a positive correlation between IL-2 levels in the CSF and the time from the onset of the first symptoms to the diagnosis of the disease in the study group. IL-2 plays a key role in stimulating the differentiation and proliferation of naïve TCD4+ lymphocytes while inhibiting differentiation towards Th17 and Tfh lymphocytes. In the context of multiple sclerosis, IL-2 contributes to both pro-inflammatory responses through the activation and expansion of autoreactive T cells and anti-inflammatory responses by promoting the development and function of Treg, which are key to maintaining immune tolerance [16].

Variable data on IL-2 levels in the CSF of patients with MS are available in the literature. Sharief et al. showed elevated levels of IL-2 in the CSF of MS patients during the active phase of the disease, which correlated with the degree of disability and the activity of flares compared to patients in remission [17]. In contrast, Ott M. et al. did not find higher levels of IL-2 in the CSF of patients with RRMS during acute relapses but noted a positive correlation between serum IL-2 levels and duration of acute relapses [18].

These variable results suggest that IL-2 levels in CSF may not be a clear marker for monitoring the progression of MS, but our results highlight the potential importance of IL-2 as an indicator of disease activity and progression in newly diagnosed RRMS cases.

Our study showed that IL-4 concentrations in the cerebrospinal fluid of patients with newly diagnosed relapsing-remitting multiple sclerosis (RRMS) were significantly higher than those of healthy controls. Furthermore, a positive correlation was found between IL-4 concentrations in cerebrospinal fluid and the time from onset of first symptoms to disease diagnosis in the study group. IL-4 is a key cytokine promoting a Th2 immune response, playing a strong anti-inflammatory role by influencing the Th1/Th2 balance in the CNS and regulating the immune response, thereby limiting tissue damage [19].

Other studies of IL-4 levels in the cerebrospinal fluid of MS patients have shown mixed results, with some suggesting that higher IL-4 levels may be associated with less active forms of the disease or with periods of remission [20,21].

Our results suggest that IL-4 may be important in the pathogenesis of RRMS, and its elevated levels may indicate disease activity and be useful in assessing disease progression.

Our results showed that patients diagnosed with de novo multiple sclerosis had lower levels of IL-5 in CSF than in the healthy population. IL-5 is a cytokine that plays a role in regulating eosinophilia activity, and its role in the pathomechanism of multiple sclerosis remains unknown [22]. Ongoing studies on the effects of glatiramer acetate (GA, Copaxon, formerly Copoly-mer-1) on IL-5 and IL-13 production in patients with multiple sclerosis have shown that those with a positive clinical response to treatment had elevated serum IL-5 and IL-13 levels [23,24].

The results presented here may indicate that IL-5 levels may be useful as a marker of response to therapy.

Analysis in this study showed that interleukin-6 (IL-6) levels in the cerebrospinal fluid of patients diagnosed with de novo multiple sclerosis (MS) were significantly higher compared to the healthy population. In addition, there was a negative correlation between IL-6 levels in CSF and serum vitamin D levels in these patients. These findings suggest that low vitamin D levels may increase inflammation, as reflected by elevated IL-6 levels in the CSF [25].

The study additionally found a positive correlation between IL-6 levels in the cerebrospinal fluid and the number of MRI lesions of the thoracic spine enhancing after contrast administration in patients diagnosed with de novo multiple sclerosis. This is likely due to the fact that IL-6, as a marker of inflammation, correlates with disease activity, as manifested by an increased number of lesions enhancing after contrast administration, an indicator of active inflammation in the central nervous system [26].

Winer et al. also demonstrated higher levels of IL-6 in the cerebrospinal fluid of patients with multiple sclerosis compared to the healthy population, further supporting these findings. In addition, available data suggest that multiple sclerosis patients with lower vitamin D levels may have higher activity and faster disease progression [27]. The results of this study highlight the importance of IL-6 as a marker of inflammation and disease activity in MS and suggest that vitamin D levels may play an important role in modulating the inflammatory response in these patients.

Our study showed that the levels of IL-7 in the cerebrospinal fluid of people with newly diagnosed relapsing-remitting multiple sclerosis (RRSM) were lower than in healthy controls.

Data from the literature on serum IL-7 concentrations in patients with multiple sclerosis are inconclusive; Haas et al. described elevated serum IL-7 levels in patients with multiple sclerosis [28], while Kreft et al. showed reduced serum IL-7 levels [29]. Our results, showing lower levels of IL-7 in the cerebrospinal fluid of patients diagnosed with de novo RRSM, may reflect specific changes at different periods of the disease and different disease activity.

These findings underscore the importance of IL-7 as a potential marker in the pathogenesis of relapsing-remitting multiple sclerosis and suggest that further studies are needed to better understand the role of IL-7 in different stages and disease activity.

Our analysis showed statistically lower levels of IL-9 in the cerebrospinal fluid of RRMS patients compared to controls. IL-9 is a cytokine that decreases and has a protective function against neurodegeneration in patients with the relapsing-remitting form of multiple sclerosis (RRMS) [30]. Similar results to ours were obtained by Ruocco et al., who found also that IL-9 levels in the cerebrospinal fluid of RRMS patients inversely correlated with the course of the disease [31]. Moreover, a study by Matsushita et al. found lower levels of IL-9 in CSF during clinical relapses and elevated values after prednisolone treatment [32]. Reduced IL-9 levels may indicate inflammation and neurodegeneration in patients with de novo RRMS. Monitoring IL-9 levels can, therefore, serve as a prognostic marker, helping to monitor the course of the disease and assess the effectiveness of therapy.

The present study showed that IL-10 levels were lower in patients with newly diagnosed RRMS compared to controls. Available literature shows reduced serum IL-10 levels in both RRMS and progressive multiple sclerosis (SPMS) patients [33]. Moreover, the anti-inflammatory role of IL-10 may be indicated by the inverse correlation we demonstrated between IL-10 levels and disease duration. This correlation may also prove chronic inflammation, which is an important factor in the pathogenesis of multiple sclerosis. Martinez-Forero et al. also observed reduced IL-10 production capacity in B lymphocytes and reduced IL-10 receptor-mediated signaling activity in TCD4+ lymphocytes in patients with multiple sclerosis [34].

Our results showed lower levels of IL-12p70 in the cerebrospinal fluid of patients with newly diagnosed RRMS, as well as a positive correlation between IL-12p70 levels and disease duration. This observation may indicate that as the disease progresses, the immune system becomes more active, leading to increased production of IL-12p70 in response to prolonged inflammation or autoimmunity. Saruhan-Direskeneli et al. demonstrated elevated levels of IL-12 in both serum and cerebrospinal fluid of MS patients but did not evaluate IL-12p70 levels separately [35]. Bartosik-Psujek and Stelmasiak found that IL-12 levels in serum and cerebrospinal fluid significantly increase during disease exacerbation, while during the stable period, they are not significantly different from those in healthy individuals [36]. The differences in the results regarding IL-12p70 levels may be due to the time of disease onset at which the studies were collected, methodological challenges, and the complex biology of IL-12.

The analysis showed higher levels of IL-13 in the cerebrospinal fluid of the study group. Similar results were obtained by Ochi et al., who showed that intracellular production of IL-13 in TCD4+ lymphocytes and TCD8+ lymphocytes of peripheral blood of patients with multiple sclerosis (MS) was significantly higher during MS remission than in healthy controls [37]. Musabak et al. also showed higher levels of IL-13 in the serum of patients with relapsing-remitting multiple sclerosis (RRMS) and isolated clinical syndrome (CIS), compared to healthy controls [38]. Higher levels of IL-13 may indicate increased inflammatory activity in patients with multiple sclerosis, especially in the early stages of the disease.

Our results showed that IL-15 levels in the group of patients with newly diagnosed multiple sclerosis were lower than in the group of healthy subjects, which differs from data available in the literature [39]. These differences may be due to the study methodology and the progression of the patient’s disease. In addition, negative correlations were found between IL-15 levels in cerebrospinal fluid and the number of T2 MRI C-spine lesions and the number of Gd(+) MRI C-spine lesions, a surprising result. IL-15, produced by monocytes and epithelial cells, stimulates the proliferation of T lymphocytes and the maturation of B lymphocytes and increases the cytotoxic capacity of NK cells, also having a chemotactic function for T lymphocytes [40].

The analysis showed lower levels of VEGF in the cerebrospinal fluid of patients with newly diagnosed RRMS. This result differs from that of Talbot et al., who showed higher levels of VEGF in the CSF of patients with the primary progressive form of multiple sclerosis (PPSM) [41]. This difference may be due to the clinical form and stage of the disease. VEGF, produced not only by immune cells but also by endothelial cells, astrocytes, and neurons, acts as a neuroprotective factor for neurons and neuronal precursors, especially in late stages of multiple sclerosis (MS) and progressive forms of the disease [42].

The analysis showed that patients with newly diagnosed multiple sclerosis had lower levels of PDGF-bb in the cerebrospinal fluid than controls, which is consistent with the literature. PDGF also stimulates remyelination of chronic lesions [43], so its reduced levels may reduce the activity of repair processes in MS patients. A positive correlation was also observed between disease duration and the number of lesions on T2 MRI of the C-spine, possibly indicating that the longer the disease duration, the greater the compensatory response. In contrast, the negative correlation of PDGF-bb levels in the cerebrospinal fluid with vitamin D is a surprising result and requires further analysis.

A study showed higher levels of GM-CSF in the cerebrospinal fluid of patients with newly diagnosed RRMS compared to healthy subjects. Similar results were obtained by Rasouli et al., who found that TCD4+ lymphocytes in MS patients showed increased GM-CSF production, while the frequency of GM-CSF-producing T cells was reduced by immunotherapy [44]. Martin et al. also demonstrated significantly higher levels of GM-CSF in the cerebrospinal fluid of MS patients, suggesting that elevated GM-CSF levels may reflect intrathecal production of this cytokine [45]. The analysis also showed that GM-CSF levels increased with the duration of symptoms, which may prove its role in the pathogenesis of MS.

The available studies focus on the use of recombinant G-CSF in the treatment of multiple sclerosis [46]. An analysis found lower G-CSF concentrations in the cerebrospinal fluid of patients with newly diagnosed multiple sclerosis than in controls. It was also observed that G-CSF levels increased with the duration of the disease, which may be the reason why these therapies did not produce the expected remission of the disease [47].

FGF-basic is a cytokine that promotes the proliferation of oligodendroglial progenitor cells (OPCs) and also contributes to angiogenesis. [48] The literature available to discuss FGF-2 concentrations has mostly focused on its serum concentration. Su et al. determined the concentration of FGF-2 in the CSF of MS patients vs. a control group of healthy subjects to show slightly elevated concentrations of FGF-2 in the CSF of the study group. [49] The results they obtained are analogous to our findings. In contrast to the study by Su et al., we showed that FGF-2 concentration increased along with the duration of the disease, while elevated FGF-2 concentration in CSF was associated with more T2 MRI spine C lesions. Su et al. showed no such correlations. [49] The above difference may be due to the different stages of the disease in patients of the study group; in our study, the patients were diagnosed de novo with MS while Su et al. investigated patients with about a nine-year history of the disease.

## 4. Materials and Methods

The study included 118 patients diagnosed with de novo multiple sclerosis (RRMS) and 112 healthy controls. Participants in both groups were inpatients at the Department of Neurology in Zabrze, between 2017 and 2022. The study group comprised individuals who met the following inclusion criteria: age > 18 years, RRMS diagnosed de novo according to the 2017 McDonald criteria [50], no earlier treatment for multiple sclerosis. The inclusion criteria for the controls were as follows: subjects with multiple sclerosis and other autoimmune co-morbidities excluded, normal MR images of the brain and spinal cord with contrast, normal findings on total CSF, and the absence of oligoclonal striations and other anibodies. Exclusion criteria for both groups were: lack of written consent to participate in the study, diagnosis of other chronic disease, other autoimmune disease, use of dietary supplements, nicotinism, alcohol abuse, and medications for chronic therapy that could significantly affect the results of the study. Patients experiencing relapse of the disease and steroid therapy were not included in the study.

Cerebrospinal fluid, 2ml, was collected in the conditions of the treatment room and then immediately after collection frozen at minus (-) 80 degrees Celsius and stored under such conditions until determinations were made.

The management of both the multiple sclerosis patients and the healthy controls was identical. We assessed the levels of selected pro-inflammatory and anti-inflammatory cytokines (IL-1β, IL-1RA, IL-2, IL-4, IL-5, IL-6, IL-7, IL-8, IL-9, IL-10, IL-12p70, IL-13, IL-15, IL-17A, TNF-α, VEGF, PDGF-bb, GM-CSF, G-CSF, and FGF-basic) using the Bio-Plex Pro Human Cytokine 27-plex Assay kit. The assays were performed according to the manufacturer’s instructions. The Bio-Plex method was used with a Bio-Plex 200 instrument (Bio-Rad, Hercules, CA, USA) [51].

CSF was collected from all the subjects and the control group, and the presence of oligoclonal striations was assessed. In addition, venous blood was collected from the subjects for routine assessment of serum vitamin D [25(OH)D] levels. The concentration was determined by chemiluminescence (Cobas 6000 apparatus) at the Laboratory of the Department of Neurology in Zabrze.

Microsoft Excel (Microsoft^®^ Excel^®^ 2019 MSO (version 2309 compilation 16.0.16827.20166) 64-bit) was used to prepare the database for calculations. Statistical analysis was performed using licensed statistical packages: Statistica v. 7.1 PL by StatSoft, MedCalc Statistical Software (MedCalc Software bvba, v.14.10.2 Ostend, Belgium), and PQStat Software v. 1.6.6. The statistical analysis assumed a level of significance (error of the first kind) of *p*(a) < 0.05 and utilized the following tests: outliers were identified and discarded using substantive analysis and the Grubbs double-sided test. Student’s *t*-test, preceded by Fisher’s F-test, was used for normally distributed variables. For unequal variances, the unequal variance t-test was applied. A Mann–Whitney U test was employed for non-normally distributed variables. The chi-square test (χ^2^) or Fisher’s exact test, with Yates correction if necessary, was used to test the independence of qualitative variables. Spearman’s rank correlation was used to analyze correlations between study variables.

Logistic regression (univariate and multivariate) was used to model the effect of study variables on a binary outcome (Control Group = 0, Study Group = 1) and estimate the Odds Ratio (OR) and ROC curve. The study was approved by the Bioethics Committee of the Silesian Medical University of Silesia in Katowice (approval no. PCN/CBN/0052/KB1/48/III/20/21/22).

## 5. Conclusions

The recent study demonstrated characteristic differences in the profiles of pro-inflammatory and anti-inflammatory cytokines in the CSF of patients with de novo diagnosed MS. In addition to evidence of the dominance of the inflammatory process in the early stages of multiple sclerosis, the study also indicates a dysregulation between the levels of pro-inflammatory and anti-inflammatory cytokines. Furthermore, the correlations between cytokine concentrations and MRI parameters, as well as vitamin D, suggest that these biomarkers may be useful in assessing disease activity and progression, as well as in monitoring the effectiveness of therapy. The interactions between these cytokines may help to better understand the mechanisms of RRMS pathogenesis and lead to a more personalized therapeutic approach. It is necessary, however, to expand the scope of the study, comprising cytokine secretion in MS patients, in particular de novo patients.

## 6. Limitations

Nevertheless, our study had also some potential limitations. The study included patients with de novo diagnosed RRMS, so the results may not be representative of patients with other forms of MS, such as primary progressive MS (PPMS) or secondary progressive MS (SPMS). The method of measurement of cytokine concentrations may be different depending on the test kits and laboratory techniques used, which may have an effect on the comparability of results with other studies.

## Figures and Tables

**Table 1:** Role of selected cytokines in the immune response [9].

Immune Response	
**Proinflammatory**	**Anti-inflammatory**
IL-1β	IL-1RA
IL-6	IL-4
IL-12	IL-10
IL-17	IL-13
TNF-α	TGF-β

**Table 2:** General characteristics of the groups.

Group	RRMS-Group Tested	Control	*p*
N ^1^	118	112	
Age [years]	38.6 ± 11.9	38.3 ± 11.9	*p* = 0.3281
Gender [% female]	73.73	79.93	*p* = 0.0841
Time from onset of first symptoms to diagnosis [years]	5.15	NA ^3^	NA
Number of T2 lesions on MRI of the brain [N]	16.65 ± 7.94	NA	NA
Number of Gd(+) lesions on MRI of the brain [N]	0.90 ± 2.73	NA	NA
Number of T2 lesions in C-spine MRI [N]	5.54 ± 6.42	NA	NA
Number of Gd(+) lesions on C-spine MRI [N]	0.35 ± 1.28	NA	NA
Number of T2 lesions in Th-spine MRI [N]	4.35 ± 5.44	NA	NA
Number of Gd(+) lesions in Th-spine MRI [N]	0.23 ± 0.42	NA	NA
Presence of oligoclonal striations type 2 in CSF [%]	74.23	NA	NA
EDSS ^2^ [score]	1.8 ± 0.6	NA	NA

^1^ Number. ^2^ Expanded Disability Status Scale. ^3^ Not applicable.

**Table 3:** Selected characteristics of descriptive statistics of cytokine concentrations in the study group and the controls with statistical analysis.

	Study Group Average Standard Deviation	Control Group Average Standard Deviation	*p*
IL-1b [pg/mL]	0.49 ±0.91	1.66 ± 2.48	*p* < 0.0001
IL-1RA [pg/mL]	5.7 ± 67.0	67.0 ± 106.3	*p* < 0.0001
IL-2 [pg/mL]	1.29 ± 0.70	1.51 ± 3.72	*p* < 0.0001
IL-4 [pg/mL]	0.23 ± 0.39	0.23 ± 0.49	*p* = 0.0065
IL-5 [pg/mL]	2.5 ± 3.1	26.9 ± 26.0	*p* < 0.0001
IL-6 [pg/mL]	8.7 ± 16.8	5.7 ± 14.2	*p* < 0.0001
IL-7 [pg/mL]	4.43 ± 3.89	7.99 ± 10.41	*p* < 0.0001
IL-8 [pg/mL]	39.9 ± 33.7	36.1 ± 22.6	*p* = 0.3584
IL-9 [pg/mL]	2.49 ± 3.38	7.26 ± 7.34	*p* < 0.0001
IL-10 [pg/mL]	0.82 ± 1.00	3.42 ± 2.26	*p* < 0.0001
IL-12p70 [pg/mL]	0.76 ± 0.76	2.42 ± 5.12	*p* < 0.0001
IL-13 [pg/mL]	5.30 ± 3.83	1.38 ± 2.32	*p* < 0.0001
IL-15 [pg/mL]	9.57 ± 5.09	161.35 ± 62.44	*p* < 0.0001
IL-17A [pg/mL]	2.56 ± 4.09	3.27 ± 6.47	*p* = 0.7889
TNFα [pg/mL]	1.87 ± 3.17	4.84 ± 16.91	*p* = 0.0524
VEGF [pg/mL]	3.43 ± 4.11	47.01 ± 27.53	*p* < 0.0001
PDGF_bb [pg/mL]	2.50 ± 9.54	17.91 ± 13.82	*p* < 0.0001
GM-CSF [pg/mL]	33.52 ± 5.86	0.91 ± 1.67	*p* < 0.0001
G-CSF [pg/mL]	3.40 ± 5.89	52.25 ± 27.87	*p* < 0.0001
FGF basic [pg/mL]	29.49 ± 12.08	13.63 ± 7.03	*p* < 0.0001

**Table 4:** Correlations of selected interleukins in CSF with serum vitamin D levels in patients with newly diagnosed RRMS and controls.

Correlated Cytokine Variables in CSF with Serum Vitamin D Levels			Study Group			Control Group		
	N	R Spearman	t(N-2)	*p*-Level	N	R Spearman	t(N-2)	*p*-Level
IL-1b [pg/mL]	70	−0.2952	−2.5482	0.0131	-	-	-	-
IL-1RA [pg/mL]	70	−0.3796	−3.3835	0.0012	-	-	-	-
IL-6 [pg/mL]	70	−0.1865	−1.9905	-0.0490	-	-	-	-
TNFα [pg/mL]	70	−0.2607	−2.2266	0.0293	-	-	-	-
PDGF-bb [pg/mL]	70	−0.2895	−2.4938	0.0151	-	-	-	-

**Table 5:** Correlations of selected interleukins in CSF with time between the onset of the first symptoms and the diagnosis.

Correlated Cytokine Variables in CSF with Disease Duration	Study Group			
	N	R Spearman	t(N-2)	*p*-Level
IL-2 [pg/mL]	103	0.2580	2.6833	0.0085
IL-4 [pg/mL]	103	0.4237	4.7012	0.0000
IL-10 [pg/mL]	103	−0.2880	−3.0222	0.0032
IL-12p70 [pg/mL]	103	0.2581	2.6853	0.0085
PDGF-bb [pg/mL]	103	0.2921	3.0699	0.0028
GM-CSF [pg/mL]	103	0.2829	2.9638	0.0038
G-CSF [pg/mL]	103	0.3106	3.2835	0.0014
FGF basic [pg/mL]	103	0.3570	3.8413	0.0002

**Table 6:** Correlations of selected interleukins in CSF with the number of T2 and Gd(+) lesions on head MRI in patients with newly diagnosed RRMS.

	Correlated Cytokine Variables in CSF with the Number of Lesions on T2 MRI of the Brain				Correlated Cytokine Variables in CSF with the Number of Gd(+) MRI Brain Lesions			
	N	R Spearman	t(N-2)	*p*-Level	N	R Spearman	t(N-2)	*p*-Level
TNFα [pg/mL]	-	-	-	-	80	0.2434	2.2165	0.0296

**Table 7:** Correlations of selected interleukins in CSF with the number of T2 and Gd(+) lesions on C-spine MRI in patients with newly diagnosed RRMS.

	Correlated Cytokine Variables in CSF with Number of Lesions on T2 MRI Spine C				Correlated Cytokine Variables in CSF with Number of Gd(+) MRI Lesions Spine C			
	N	R Spearman	t(N-2)	*p*-Level	N	R Spearman	t(N-2)	*p*-Level
IL-15 [pg/mL]	80	−0.2461	−2.2420	0.0278	80	−0.2730	−2.5058	0.0143
TNFα [pg/mL]	80	0.2434	2.2165	0.0296	-	-	-	-
PDGF_bb [pg/mL]	80	0.2218	2.0093	0.0480	-	-	-	-
FGF basic [pg/mL]	80	0.2201	1.9927	0.0498	-	-	-	-

**Table 8:** Correlations of selected interleukins in CSF with the number of T2 and Gd(+) lesions on Th spine MRI in patients with newly diagnosed RRMS.

	Correlated Cytokine Variables in CSF with Number of Lesions on T2 MRI of Th Spine				Correlated Cytokine Variables in CSF with Number of Gd(+) MRI Lesions of Th Spine			
	N	R Spearman	t(N-2)	*p*-Level	N	R Spearman	t(N-2)	*p*-Level
IL-6 [pg/mL]	-	-	-	-	13	0.5863	2.4007	0.0352

## Data Availability

The dataset obtained in the research is available from the corresponding author on a reasonable request.
